# Tick-borne encephalitis in Europe, 2012 to 2016

**DOI:** 10.2807/1560-7917.ES.2018.23.45.1800201

**Published:** 2018-11-08

**Authors:** Julien Beauté, Gianfranco Spiteri, Eva Warns-Petit, Hervé Zeller

**Affiliations:** 1 European Centre for Disease Prevention and Control (ECDC), Stockholm, Sweden; 2 European Centre for Disease Prevention and Control (ECDC), Stockholm, Sweden (affiliation when the work was performed); 3 Direction Départementale de la Cohésion Sociale et de la Protection des Populations d’Ille-et-Vilaine, Rennes, France (current affiliation)

**Keywords:** Europe, epidemiology, tick-borne encephalitis (TBE), surveillance, tick-borne diseases, vector-borne infections, viral encephalitis

## Abstract

Since 2012, tick-borne encephalitis (TBE) is a notifiable in the European Union. The European Centre for Disease Prevention and Control annually collects data from 28 countries plus Iceland and Norway, based on the EU case definition. Between 2012 and 2016, 23 countries reported 12,500 TBE cases (Ireland and Spain reported none), of which 11,623 (93.0%) were confirmed cases and 878 (7.0%) probable cases. Two countries (Czech Republic and Lithuania) accounted for 38.6% of all reported cases, although their combined population represented only 2.7% of the population under surveillance. The annual notification rate fluctuated between 0.41 cases per 100,000 population in 2015 and 0.65 in 2013 with no significant trend over the period. Lithuania, Latvia and Estonia had the highest notification rates with 15.6, 9.5 and 8.7 cases per 100,000 population, respectively. At the subnational level, six regions had mean annual notification rates above 15 cases per 100,000 population, of which five were in the Baltic countries. Approximately 95% of cases were hospitalised and the overall case fatality ratio was 0.5%. Of the 11,663 cases reported with information on importation status, 156 (1.3%) were reported as imported. Less than 2% of cases had received two or more doses of TBE vaccine.

## Background

Tick-borne encephalitis (TBE) is an infectious disease of the central nervous system caused by a flavivirus and usually transmitted by the bite of infected *Ixodes* spp. These ticks can be found from western Europe to Japan [1]. Less frequently, humans can be infected by drinking contaminated milk. Many vertebrate species can be infected by the TBE virus but ticks are the main reservoir for the virus. There are three subtypes of the TBE virus: the European subtype (TBEV-Eu) is mainly transmitted by *I. ricinus* while both the Far-eastern (TBEV-FE) and Siberian (TBEV-Sib) subtypes are mainly transmitted by *I. persulcatus*. Recent findings from Finland suggest that *I. ricinus* can also transmit TBEV-Sib [2]. In Europe, most cases are infected by TBEV-Eu but cases infected with TBEV-FE were reported in Estonia and Latvia [1] and with TBEV-Sib in Estonia [3] and Finland [4].

The typical course of the disease is biphasic. After a median incubation period of 8 days, the first stage consists of a few days of non-specific symptoms such as fever, fatigue and body pain. After a symptom-free week, approximately one-third of infected persons can develop neurological conditions [5], ranging from mild meningitis to severe encephalitis [1]; increasing age is a known risk factor for severe TBE. Infection with TBEV-FE is associated with more severe disease with case fatality as high as 20–40% compared with 1–2% with TBEV-Eu [6].

There is no curative treatment for TBE but a vaccine is available. This vaccine is highly immunogenic [7] and the impact of mass vaccination in Austria is suggestive of good effectiveness [8]. Vaccine schedules for the two vaccines licensed in Europe based on TBEV-Eu strains require three doses followed by boosters [1]. In a position paper on TBE vaccination published in 2011, the World Health Organization (WHO) recommended that TBE vaccination should be offered to all age groups in highly endemic areas (i.e. areas with TBE incidence above 5 cases per 100,000 population) [9].

In Europe, most cases occur during June-September [10]. *Ixodes* spp. are found in large parts of Europe but areas at risk for TBE are mainly located in central and eastern Europe and the Baltic and Nordic countries [11]. Between 2000–2010, the annual number of TBE cases reported in the European Union and European Economic Area (EU/EEA) fluctuated between 2,000–3,500 cases [11,12]. Spikes in cases of TBE have occurred in some years, e.g. 2006, but this was likely a result of changes in human behaviour based on suitable weather conditions (e.g. increased outdoor recreational activities) [13]. More recently, some countries, e.g. Belgium and the Netherlands, reported possible new endemic foci having found antibodies to the TBE virus in roe deer and cattle [14,15] and in 2016, the Netherlands reported their first locally-acquired human case [16]. The mapping of endemic foci is essential to make recommendations for vaccination programme and travel advice [17]. In 2011, the first attempt to collect TBE surveillance data at the EU/EEA level underlined the need for an agreed case definition and systematic data collection [11]. Therefore, in 2012, the European Commission included TBE in the list of notifiable diseases in the EU/EEA [17].

Here, we describe TBE cases reported in the EU/EEA between 2012 and 2016.

## Methods

Since 2012, the European Centre for Disease Prevention and Control (ECDC) requires all 28 EU Member States, plus Iceland and Norway, to annually report their TBE data to the European Surveillance System (TESSy) database using the EU case definition (Box) [18]. More detailed information on surveillance systems is available elsewhere [10]. We included all cases reported during the years 2012–2016 meeting the EU case definition in the analysis.

Box **European Union case definition for tick-borne encephalitis**

A confirmed case is defined as any person meeting the clinical criteria i.e. symptoms of inflammation of the central nervous system

AND

• Has laboratory-confirmation i.e. at least one of the following five:

• Tick-borne encephalitis (TBE) specific IgM and IgG antibodies in blood.

• TBE specific IgM antibodies in cerebrospinal fluid.

• Sero-conversion or fourfold increase of TBE-specific antibodies in paired serum samples.

• Detection of TBE viral nucleic acid in a clinical specimen.

• Isolation of TBE virus from clinical specimen.

A probable case is defined as any person meeting the clinical criteria and the laboratory criteria for a probable case i.e. detection of TBE-specific IgM-antibodies in a unique serum sample

OR

Any person meeting the clinical criteria with exposure to a common source (unpasteurised dairy products).

TBE Information received included age, sex, date of disease onset, probable place of infection, place of residence, importation status, hospitalisation status, vaccination status, and clinical outcome. Coded values for variables with geographical information (probable place of infection and place of residence) followed the nomenclature of territorial units for statistics (NUTS) of the EU [19].

We used population denominator data provided by the Statistical Office of the EU (Eurostat) for calculating rates (data extracted on 22 September 2017). We compared continuous variables by the Mann–Whitney U test and categorical variables using the chi-squared test. We estimated annual rates of change and their 95% confidence intervals (CI) using a log-linear regression of notification rates over the period 2012–2016. We assessed goodness of fit of linear regressions using F statistics. We used Stata software release 14 (StataCorp. LP, United States) for all data management and statistical analyses.

## Results

### Case classification and notification rate

Over the 2012–2016 period, 23 countries reported 12,500 TBE cases (Ireland and Spain reported no cases), of which 11,622 (93.0%) were confirmed cases and 878 (7.0%) probable cases (Table 1). We excluded 31 cases with unknown classification (11 cases for Austria, 15 cases for Lithuania, four cases for Poland and one case for Slovenia). Cyprus, Iceland, Malta, and Portugal had no TBE surveillance and Denmark did not report any data.

**Table 1:** Number of reported cases^a^ of tick-borne encephalitis, percentage of imported cases, notification rate per 100,000 population and trend, in 25 countries, European Union and European Economic Area, 2012–2016 (n = 12,500)

Country	2012		2013		2014		2015		2016		Total cases			Trend	
	Number of cases	Notification rate	Number of cases	Notification rate	Number of cases	Notification rate	Number of cases	Notification rate	Number of cases	Notification rate	Number of cases	Imported cases n (%)^b^	Notification rate	Annual variation (%)	95% CI
Austria	38	0.45	100	1.18	81	0.95	79	0.92	95	1.09	393	6 (1.7)	0.92	15.2	- 19.6 to 49.9
Belgium	2	0.02	1	0.01	0	0.00	1	0.01	1	0.01	5	3 (100)	0.01	NA	NA
Bulgaria					0	0.00	2	0.03	0	0.00	2	Unknown	< 0.01	NA	NA
Croatia	45	1.05	44	1.03	23	0.54	26	0.62	6	0.14	144	Unknown	0.68	- 45.1	- 90.5 to 0.3
Czech Republic	573	5.45	625	5.94	410	3.90	349	3.31	565	5.35	2,522	16 (0.6)	4.79	- 6.2	- 33.0 to 20.6
Estonia	178	13.43	114	8.64	83	6.31	116	8.82	81	6.16	572	2 (0.3)	8.68	- 15.4	- 39.0 to 8.2
Finland	39	0.72	38	0.70	47	0.86	68	1.24	61	1.11	253	Unknown	0.93	14.4	0.7 to 28.1
France	1	< 0.01	2	< 0.01	9	0.01	10	0.02	22	0.03	44	9 (21.4)	0.01	77.3	42.0 to 112.7
Germany	195	0.24	419	0.52	264	0.33	219	0.27	348	0.42	1,445	47 (3.5)	0.36	4.6	- 31.1 to 40.2
Greece	0	0.00	0	0.00	1	0.01	1	0.01	0	0.00	2	0	< 0.01	NA	NA
Hungary	44	0.44	53	0.53	31	0.31	24	0.24	19	0.19	171	2 (1.2)	0.35	- 24.5	- 42.9 to - 6.1
Ireland	0	0.00	0	0.00	0	0.00	0	0.00	0	0.00	0	0	0	NA	NA
Italy			0	0.00	0	0.00	5	0.01	48	0.08	53	3 (6.3)	0.02	NA	NA
Latvia	229	11.20	230	11.36	149	7.44	141	7.10	204	10.36	953	0	9.51	- 6.3	- 30.2 to 17.7
Lithuania	494	16.45	487	16.39	353	11.99	336	11.50	633	21.91	2,303	18 (0.9)	15.64	2.2	- 28.3 to 32.7
Luxembourg	NA	NA	NA	NA	0	0.00	1	0.18	0	0.00	1	1 (100)	0.06	NA	NA
The Netherlands	NA	NA	NA	NA	NA	NA	NA	NA	4	0.02	4	2 (50.0)	0.02	NA	NA
Norway	7	0.14	6	0.12	13	0.25	9	0.17	12	0.23	47	7 (15.9)	0.18	13.7	- 13.9 to 41.3
Poland	187	0.49	226	0.59	195	0.51	149	0.39	283	0.75	1,040	8 (0.8)	0.55	4.2	- 22.4 to 30.7
Romania	3	0.01	3	0.01	1	0.01	0	0.00	0	0.00	7	0	< 0.01	NA	NA
Slovakia	102	1.89	163	3.01	116	2.14	84	1.55	173	3.19	638	5 (0.8)	2.36	3.8	- 31.2 to 38.9
Slovenia	164	7.98	307	14.91	100	4.85	62	3.01	83	4.02	716	1 (0.2)	6.95	- 29.7	- 78.6 to 19.2
Spain	0	0.00	0	0.00	0	0.00	0	0.00	0	0.00	0	0	0	NA	NA
Sweden	287	3.03	209	2.19	178	1.85	268	2.75	238	2.42	1,180	21 (1.8)	2.44	- 2.2	- 24.3 to 19.9
UK	3	< 0.01	0	0.00	2	< 0.01	0	0.00	0	0.00	5	5 (100)	< 0.01	NA	NA
**EU/EEA**	**2,591**	**0.64**	**3,027**	**0.65**	**2,056**	**0.43**	**1,950**	**0.41**	**2,876**	**0.58**	**12,500**	**156 (1.3)**	**0.54**	**- 6.6**	**- 29.1 to 16.0**

CL: confidence interval; EU/EEA: European Union and European Economic Area; NA: not available; UK: United Kingdom.

^a ^Number of cases includes both confirmed and probable cases.

^b^ cases with known importation status.

Most countries (18/23) reported over 90% of cases as confirmed. Slovakia (552/638; 86.5%), France (36/44; 81.8%), Hungary (131/171; 76.6%), Latvia (683/953; 71.7%), and Poland (712/1,040; 68.5%), classified the lowest proportions of their cases as confirmed. The mean annual notification rate was 0.54 cases per 100,000 population.

### Importation

Of the 11,664/12,500 cases reported with information on importation status, 156 (1.3%) were reported as imported (Table 1). Importation status was missing for cases reported by Bulgaria, Croatia, and Finland. All cases reported in Belgium, Luxembourg, and the United Kingdom (UK) were imported. Information on the probable country of infection was available for 152 of these imported cases (97.4%). Top destinations for travel-associated TBE were Austria (32 cases, 21.1% of all imported cases), Sweden (19 cases, 12.5%) and Finland (18 cases, 11.8%). Four countries (the Czech Republic, Germany, Lithuania, and Sweden) reported 102/156 (65.3%) of all imported cases. Imported cases were slightly younger than locally-acquired cases (median age for imported cases: 46 years; locally-acquired cases: 48 years; p = 0.03) and more likely to be male (imported cases: 71% males; locally-acquired cases: 59% males; p < 0.01).

### Geographical distribution

**Figure 1:**
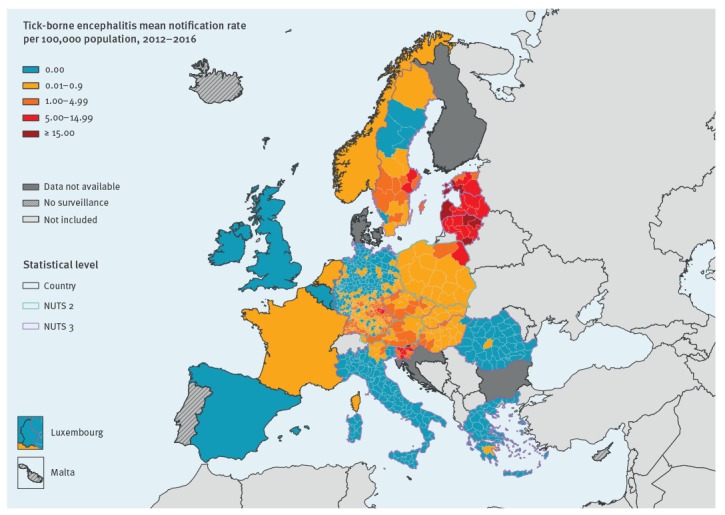
Rate of locally acquired tick-borne encephalitis per 100,000 population, by place of infection, European Union and European Economic Area countries, 2012–2016

Two countries (Czech Republic and Lithuania) accounted for 4,825/12,500 (38.6%) of all reported cases (Table 1). Of the 23 countries that reported cases, 16 had mean notification rates below one case per 100,000 population. Over the 2012–2016 period Lithuania, Latvia and Estonia had the highest notification rates with 15.6, 9.5 and 8.7 cases per 100,000 population, respectively (Table 1 and Figure 1). Among the 23 countries that reported cases, 17 had locally-acquired cases. Of these, 12 provided geographical information at NUTS3 level, two at NUTS2 (Austria and Poland), and three did not have information at subnational level (France, the Netherlands, and Norway) (Figure 1). At the subnational level, six regions had mean annual notification rates above 15 cases per 100,000 population: Utena county, Lithuania (44.5), Lääne-Eesti, Estonia (27.7), Kurzeme, Latvia (23.8), Alytus county, Lithuania (22.1), Panevėžys county, Lithuania (19.1) and Carinthia, Slovenia (15.1) (Figure 1).Twenty-nine regions in seven countries (Estonia, Germany, Latvia, Lithuania, Poland, Slovenia and Sweden) had notification rates above five cases per 100,000 population. In Lithuania, Telšiai County was the region with the lowest mean annual notification rate (5.6).

ECDC. Map produced on: 19 July 2018

### Trend and Seasonality

The overall annual notification rate fluctuated between a minimum of 0.41 cases per 100,000 population in 2015 and a maximum of 0.65 in 2013 with no significant trend over the period (annual variation of - 6.6% (95% CI: - 29.1 to 16; p = 0.4) (Table 1). We observed significant trends for three countries: the TBE notification rate increased at an annual rate of 14.4% (95%CI: 0.7 to 28.1) in Finland and 77.3% (95%CI: 42.0 to 112.7) in France and decreased at an annual rate of 24.5% (95%CI: 6.1 to 42.9) in Hungary.

**Figure 2:**
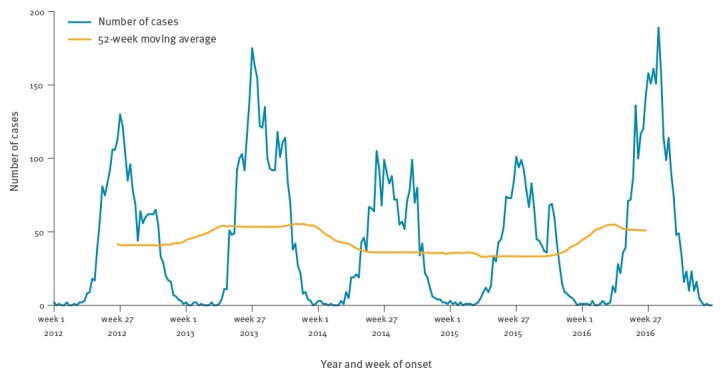
Number of reported tick-borne encephalitis cases by month of onset, and 12-month moving average, 19 European Union and European Economic Area countries, 2012–2016

**Figure 3:**
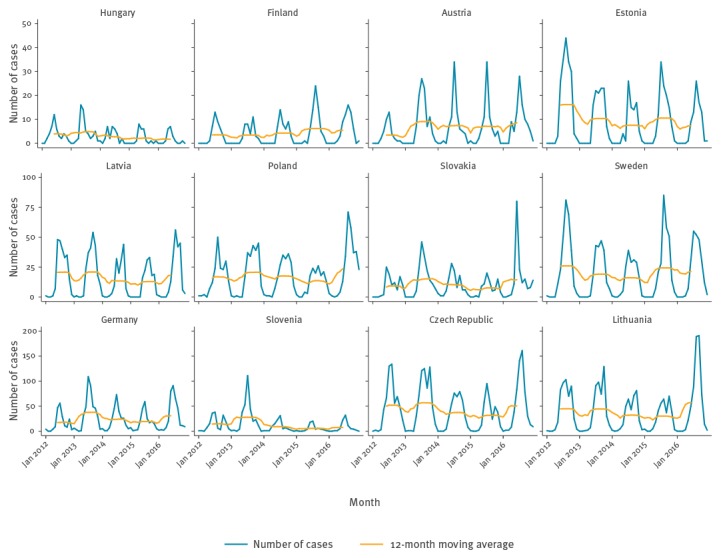
Number of reported tick-borne encephalitis cases by week of onset and 52-week moving average, 12 European Union and European Economic Area countries, 2012–2016

Of the 11,397 cases reported with onset date, 10,632 (93.3%) had an onset month May–October and 135 (1.2%) had an onset month December–March (off-season) (Figure 2). We observed a comparable seasonality in the 12 countries reporting at least 100 cases over the period with onset month (Figure 3). There were peaks in 2012 (Estonia and Sweden), 2013 (Germany, Hungary, Slovakia, Czech Republic and Slovenia), 2014 (Austria), 2015 (Austria, Estonia, Finland, Sweden), and 2016 (Czech Republic, Germany, Lithuania, Poland and Slovakia).

Includes data from: Austria, Czech Republic, Estonia, France, Germany, Greece, Hungary, Italy, Latvia, Latvia, Lithuania, Luxembourg, Netherlands, Norway, Poland, Romania, Slovakia, Slovenia and Sweden.

### Demographics

**Figure 4:**
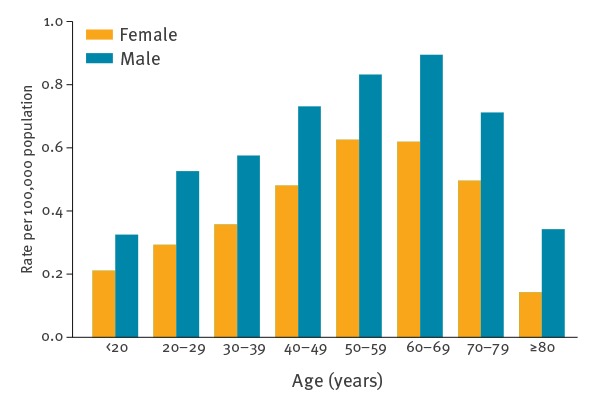
Notification rates of tick-borne encephalitis per 100,000 population, by sex and age group and male-to-female rate ratio by age group, European Union and European Economic Area countries, 2012–2016

Of the 12,470 cases reported with information on age, 6,782 (54.4%) were in the 40–69 years old group (Table 2). TBE was more common in males with a male-to-female rate ratio of 1.5:1. Notification rates increased with age in both sexes, peaking at 0.89 cases per 100,000 population in males aged 60–69 years, and then decreased in older age groups (Figure 4). At date of disease onset, females (median 51 years, interquartile ratio (IQR): 35–62) were older than males (median 47 years, IQR: 31–61) (p < 0.01).

**Table 2:** Main characteristics of reported cases of tick-borne encephalitis, European Union and European Economic Area countries, 2012−2016 (n = 12,500)

Characteristics	Number of cases	Percent	Notification rate per 100,000 persons
**Total**	**12,500**	**100**	**0.54**
**Age group (years)**			
< 20	1,402	11.2	0.29
20–29	1,257	10.1	0.44
30–39	1,575	12.6	0.50
40–49	2,200	17.6	0.65
50–59	2,474	19.8	0.77
60–69	2,108	16.9	0.80
70–79	1,183	9.5	0.63
≥ 80	271	2.2	0.23
Unknown	30	NA	NA
**Sex**			
Female	5,118	40.9	0.43
Male	7,381	59.1	0.65
Unknown	1	NA	NA
**Importation status**			
Imported	156	1.3	NA
Locally-acquired	11,507	98.7	NA
Unknown	837	NA	NA
**Hospitalisation**			
Yes	7,672	94.9	NA
No	409	5.1	NA
Unknown	4,419	NA	NA
**Outcome**			
Alive	9,594	97.0	NA
Dead	48	0.5	NA
Neurological complications	247	2.5	NA
Unknown	2,611	NA	NA
**Vaccination status**			
Four doses	24	0.5	NA
Three doses	36	0.7	NA
Two doses	27	0.5	
One dose	33	0.6	NA
Vaccinated unknown doses	19	0.4	NA
Not vaccinated	5,066	97.3	NA
Unknown	7,295	NA	NA

NA: not available.

### Outcome

Of the 8,081 cases reported with hospitalisation status, 7,672 (94.9%) were admitted to hospital (Table 2). Of the 9,889 cases reported with known outcome, 48 (0.5%) died and 247 (2.5%) had neurological sequelae. The case fatality ratio did not differ significantly by sex (0.5% in males vs 0.4% in females, p = 0.30). The case fatality ratio was higher in older age groups (3.1% in cases aged 80 years or older, 2.0% in cases aged 70–79 years and < 0.5% in cases aged below 70 years).

### Vaccination

Of the 5,205 cases with known vaccination status, 5,066 (97.3%) were not vaccinated, 60 had received one or two doses (1.2%), 60 (1.2%) three doses or more and 19 (0.4%) an unknown number of doses (Table 2). Of the 20 cases with fatal outcome and known vaccination status, 19 were not vaccinated and one had received one dose of the vaccine. The proportion of cases that received two doses or more of vaccine was higher in the extreme age groups compared with the other groups (2.5% in both cases aged 20 years or younger and 70 years or older). No imported cases had received more than one vaccine dose.

## Discussion

The European TBE surveillance data suggest a stable trend over the years 2012–2016 with no reported changes in national surveillance systems; continuing the long-term trend observed in Europe since the mid-1990s [12]. The number of TBE cases reported in Europe, excluding Russia, increased over the years 1990–1994, probably reflecting the start of surveillance in many countries [12]. Over the following 15 years (1995–2009), the trend was stable with an annual number of TBE cases fluctuating between 2,000 and 4,000 cases. Peaks occurred when a set of countries reported unusually high numbers of TBE cases, e.g. 2006 and 2009 [12]. In 2013, several European countries experienced a peak in TBE cases, which resulted in the highest number of TBE cases (> 3,000) observed in Europe that year. An analysis carried out in eight European countries suggested that human behaviour in response to good weather conditions, e.g. increased outdoor recreational activities, was the main explanation for the 2006 spike rather than tick abundance [13].

The overall stable trend observed in TBE surveillance data is mainly driven by a few countries reporting the majority of cases, potentially masking important disparities both between and within countries. For example, two countries (Czech Republic and Lithuania) accounted for 38.6% of all reported TBE cases, although their combined population represented only 2.7% of the population of the 25 countries included in this analysis. All countries with average annual notification rate above one TBE case per 100,000 population had a stable trend over the period. We only observed an increase in Finland and France. In France, the notification rate almost tripled in 2016 compared with previous years in the Alsace region where most cases occurred [20]; some newly identified foci such as the Alpine region could also have contributed to the upsurge in cases. However, the reasons behind this increase are yet to be determined. In Finland, the emergence of new foci reported during 1995–2013 could partly explain the increase [21]. A decrease in TBE cases was observed in Hungary over the years 2012–2016, to our knowledge there is no explanation as to why. Trends at country level, such as these, may mask changes at local level as TBE endemicity is very focal and countries do not have a uniform risk across all territories/regions/counties etc. In Lithuania, which had the highest average annual notification rate, there was an eightfold difference between counties with highest and counties lowest TBE incidence. An analysis of epidemiological patterns of TBE in Lithuania suggested different trends across counties with more pronounced increases in eastern and northern parts of the country [22]. Similarly, diverging trends across regions were reported in Austria [23]. Decreasing trend were observed in north-east of Lower Austria whilst the alpine regions in the west of Austria became highly endemic.

Independently of what happens in animal reservoirs, we can classify factors driving TBE incidence in three groups: (i) tick abundance, (ii) population at risk, (iii) surveillance characteristics. Factors related to tick abundance are multiple (e.g. land, weather, reservoirs etc.) and can be very focal. The impact of climate change is debated with possibly different effects in different settings. A study carried out in Sweden suggested that milder winters were associated with increased TBE incidence in the mid-1980s [24]. Yet, a general circulation model predicted that TBE transmission could be disrupted by climate change with a contraction of TBE areas to higher altitudes in central Europe and northern latitudes in Scandinavia [25]. This would result in a decreased incidence in the coming decades but such change would probably not be captured over a 5–year period. Changes in human behaviour (e.g. increase of at-risk outdoor recreational activities) can put people at greater risk of exposure to ticks and thus TBE. However, with increased vaccine coverage such risk could be improved. Finally, better clinical awareness, testing and reporting would improve the ability of the surveillance system to detect cases.

The geographical granularity of our data (at best NUTS3) does not allow fine monitoring of TBE foci, which countries are best placed to perform. However, during the first effort to collect TBE data at the EU/EEA level most of the recommendations were followed [11]. We implemented standard EU case definition for TBE and initiated routine collection of surveillance data from EU/EEA countries, to at least NUTS-3 geographical level for most of the countries. ECDC encourages all countries to report their cases at subnational level.

The reported TBE cases followed a pronounced seasonality with most cases occurring during the warmer months May–October, which is likely due to human habits with people spending a greater amount of time outdoors in areas e.g. forests where ticks populations are high [1]. Cases infected during colder months are possible, however, especially in central Europe.

Cases of TBE are more common in older age groups, with the highest number of cases occurring in those aged 40-69 years. The highest notification rate, in those aged 60-69 years, most likely reflects high exposure to tick populations at an age where individuals have increased time for outdoor recreational activity, but also fall into the known higher severity seen in older age groups [26].

Almost 95% (7,672/8,081) of reported TBE cases were admitted to hospital, which is not unexpected given that the clinical criteria used in the case definition selects severe cases. Even though the overall case fatality was relatively low, it was far from negligible in older age groups at ca 2–3% above 70 years of age. Previous reviews suggested that a third of patients could suffer long-lasting sequelae [1]. Our analysis found a much lower proportion but it is likely that our data could not capture long-term sequelae that would be diagnosed later in time from acute infection and thus not reported.

Vaccination remains the most effective protective measure against TBE [27]. However, studies have reported vaccine failures, especially in older age groups [28]. We found that 87/5,205 (1.7%) of cases were supposedly vaccinated (at least two doses of vaccine), mostly in extreme age groups. This would be in line with results from studies suggesting that age and number of vaccine doses were the most important factors determining the immunological response to vaccination [29]. The extended period between doses may mean that people are less likely to comply to the recommendations as shown in Germany where compliance after the first dose was low [30]. Another reason for not receiving or completing TBE vaccination is cost. TBE vaccination is not reimbursed in most EU/EEA countries and the willingness to pay for vaccination may not be sufficient to ensure uptake in residents or visitors frequenting areas considered high risk for tick populations and TBE [31]. A survey published in 2008, reported that Austria, Finland, Germany, Hungary, Latvia and Slovenia included TBE in their routine vaccination programme at least for some specific groups or areas [32].

In this study, we only found a few TBE cases in international travellers. Cases that are resident in countries with little or no risk of TBE are less likely to be vaccinated or diagnosed [33]. Increased awareness of TBE is required to improve vaccination coverage in travellers and promote the best practices to avoid tick bites. Currently, the WHO recommends vaccination of travellers who are at risk of TBE exposure during outdoors activities in rural endemic areas during the period of transmission [34].

## Conclusion

The overall TBE notification rate remained stable during 2012–2016. Surveillance at EU/EEA level helped provide reliable and comparative data allowing better mapping of the disease risk both at the national and subnational level. Countries with regions where the disease is highly endemic should consider strengthening information campaigns on preventive measures against tick bites as well as introducing TBE vaccine recommendations if these are not already proposed. ECDC encourages countries to report better quality and more complete data on TBE diagnoses, particularly on the sub-national geographic distribution and on imported cases.
